# What Are “Bioplastics”? Defining Renewability, Biosynthesis, Biodegradability, and Biocompatibility

**DOI:** 10.3390/polym15244695

**Published:** 2023-12-13

**Authors:** Maximilian Lackner, Anindya Mukherjee, Martin Koller

**Affiliations:** 1 Go!PHA, Oudebrugsteeg 9, 1012 JN Amsterdam, The Netherlands; anindya.mukherjee@gopha.org; 2 Go!PHA, 12324 Hampton Way, Wake Forest, NC 27587, USA; 3 CIRCE Biotechnologie GmbH, Kerpengasse 125, 1210 Vienna, Austria; 4 Institute of Chemistry, NAWI Graz, University of Graz, Heinrichstrasse 28/IV, 8010 Graz, Austria; martin.koller@uni-graz.at

**Keywords:** biobased carbon content, biodegradability, aerobic, anaerobic, biopolymer, bioplastics, composite, composting, marine, litter, EN 13432, bio-attributed, renewable carbon, circular economy, degradation, renewable, circular

## Abstract

Today, plastic materials are mostly made from fossil resources, and they are characterized by their long lifetime and pronounced persistence in the open environment. These attributes of plastics are one cause of the ubiquitous pollution we see in our environment. When plastics end up in the environment, most of this pollution can be attributed to a lack of infrastructure for appropriately collecting and recycling plastic waste, mainly due to mismanagement. Because of the huge production volumes of plastics, their merits of being cheap to produce and process and their recalcitrance have turned into a huge disadvantage, since plastic waste has become the end point of our linear economic usage model, and massive amounts have started to accumulate in the environment, leading to microplastics pollution and other detrimental effects. A possible solution to this is offered by “bioplastics”, which are materials that are either (partly) biobased and/or degradable under defined conditions. With the rise of bioplastics in the marketplace, several standards and test protocols have been developed to assess, certify, and advertise their properties in this respect. This article summarizes and critically discusses different views on bioplastics, mainly related to the properties of biodegradability and biobased carbon content; this shall allow us to find a common ground for clearly addressing and categorizing bioplastic materials, which could become an essential building block in a circular economy. Today, bioplastics account for only 1–2% of all plastics, while technically, they could replace up to 90% of all fossil-based plastics, particularly in short-lived goods and packaging, the single most important area of use for conventional plastics. Their replacement potential not only applies to thermoplastics but also to thermosets and elastomers. Bioplastics can be recycled through different means, and they can be made from renewable sources, with (bio)degradability being an option for the mismanaged fraction and special applications with an intended end of life in nature (such as in seed coatings and bite protection for trees). Bioplastics can be used in composites and differ in their properties, similarly to conventional plastics. Clear definitions for “biobased” and “biodegradable” are needed to allow stakeholders of (bio)plastics to make fact-based decisions regarding material selection, application, and end-of-life options; the same level of clarity is needed for terms like “renewable carbon” and “bio-attributed” carbon, definitions of which are summarized and discussed in this paper.

## 1. Introduction

**Figure 1:**
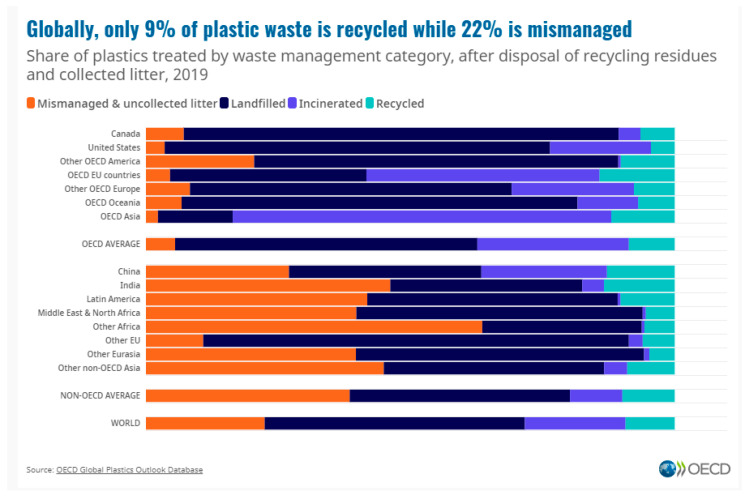
Mismanagement of plastics is at the core of plastic-related environmental problems. Reproduced with permission from [3].

Plastics are a versatile group of materials with countless short-lived and durable applications. This versatility is a result of the possibility to produce polymers with different chain lengths and molecular weight distributions from various monomers, and from the ability to derive composites, e.g., through the addition of fillers that modify their mechanical properties over a wide range. Examples of articles with a short service lifetime are food and non-food packaging, while examples of articles with long lifetimes are plastic window frames and plastic pipes for drinking water, sewage, and natural gas. In some applications, alternatives already exist (e.g., glass or metal packaging), while for other cases, no practical alternatives or substitutes are in place, e.g., for electrical applications, where the unique properties of plastics (e.g., their high thermal and high electrical insulation, light weight, low cost per item, durability, and resistance against various media) necessitate their use. It is hard to imagine our daily life without plastics. The annual plastic production volume was 390.7 million tons in 2021 [1], 90.2% of which were virgin fossil-derived polymers, with only 8.3% being post-consumer recycled plastics and approx. 1.5% being renewable or “bio-based/bio-attributed” plastics [1]. Overall, recycled plastic materials accounted for 11.7% of all plastics in Europe [2], a figure that has gone up by only 1% during the last decade. Without drastic changes in our use and reuse patterns, the path to proclaimed “circularity” is out of reach. At a global level, no more than 9% of all plastics are recycled [3]. The major issue with plastics waste is the mismanagement of plastic items towards and at the end of their lifetime, which can be attributed to a lack of waste collection and recycling infrastructure in most developing and emerging countries and inadequate or inappropriate waste collection and sorting infrastructure even in the developed/industrialized countries, coupled with human behavioural aspects, and the leakage of micro- and nanoplastics particles that are hard-to-impossible to catch and retain with today’s technologies. Also, the broad range of plastic types and compounds, with different colors, fillers, and additives, aggravates the difficulties in attempts at (mechanical) recycling. Of note, the availability and price of raw fossil plastics are often more advantageous than those of recycled plastics, due to the low prices of fossil plastics (particularly commodities such as poly(ethene) (PE), poly(propene) (PP), poly(vinyl chloride) (PVC), and poly(ethene terephthalate) (PET)). Also, the demand for recycled plastics has increased strongly in some areas, with “green” products being more accepted by the customers, leading to comparatively higher prices and some shortages in supply. Figure 1 shows that globally, the amount of mismanaged materials is more than double the amount of recycled plastics.

The use of plastics largely follows a linear system (the “take-use-dispose” paradigm). A very recent report by the UN states [4]:

- Up to 80% of mismanaged plastics could be reduced by 2040.
- The annual costs from mismanaged plastics are 300–600 billion USD.

Simply improving today’s recycling practices will not suffice. For instance, it was found that a single plastic waste recycling company in the UK emits up to 1.5 million kg of microplastics per year [5], which is half of the entire quantity of microplastics generated by this company; hence, half of the produced microplastics cannot be retained via filters or other means and enter the environment. In general, the formation and emission of microplastics has been undervalued and only recently has awareness on the topic emerged. Several companies are already developing solutions for capturing microplastics at the source, e.g., behind car tires or in washing machines; however, such attempts cannot catch the entire micro- and nanoplastics freight, and most points where microplastics occur, such as at sewage treatment plants, are not equipped with capturing technology at all. The European plastics industry has launched an effort called operation clean sweep (OCS) to reduce the emission (spills and losses) of plastics pellets, flakes and powder throughout the value chain, which constitute another major source of microplastics entering the environment. Also, technologies are being developed to capture bulk plastic items already littered into water bodies, e.g., via bubble curtains [6], yet all such attempts are at the very beginning and far from large-scale roll-out.

In order to reach recycling rates for plastics that are comparable to those of wood, paper, glass, and metals, paradigm changes are needed, with concerted action in legislation and technology development required, accompanied by changes in people’s attitude towards plastics and inadvertent plastics “dumping”.

Plastics are a virtually indispensable class of materials, and they have a bad reputation amongst consumers as numerous studies show, e.g., a recent one where consumers reported viewing plastics as the least environmentally friendly packaging material [7]. It seems that intrinsic material properties are mixed with plastics’ mismanagement and its consequences, but the root causes for that problem are hardly known and hence not addressed. To stick with that example, plastic materials can offer several advantages over other packaging materials, and a key question is how to make them circular, less carbon-intense, and, in general, more sustainable. According to the OECD, “sustainable plastics” can be defined as “*plastics used in products that provide societal benefits while enhancing human and environmental health and safety across the entire product life cycle*” [8].

This article focuses on the definitions of bioplastics alongside the dimensions of being “biobased” and/or being “biodegradable”. There is no plain “black and white” here, with many misconceptions. Therefore, this article intends to provide a detailed overview on bioplastics, and then delves into existing definitions. The key novelty and unique aspect of this review lies in offering an up-to-date summary of existing bioplastics definitions, with the aim of providing readers with a fast and handy reference.

## 2. State of the Art in Bioplastics

A “plastic” by definition is a polymer-based formulation, which consists of one or more polymers (homopolymer, copolymers, blends) plus additives and fillers. In nature, several polymers can be found, e.g., starch, cellulose, lignocellulose, or proteins (so-called biopolymers and/or naturally occurring polymers). A bioplastic (bioplastics) can be defined as a biopolymer-derived formulation, e.g., starch + plasticizer, poly(lactic acid) (PLA) + additives for processing and coloration, or (natural) fiber-reinforced poly(3-hydroxybutyrate) (P3HB), to give three well-established examples. A plastic material derives its properties from the combination of polymer(s) and additives, which applies equally to fossil and to biobased plastics. Filled products are called “compounds” or “composite materials”.

### 2.1. What Are Bioplastics?

**Figure 2:**
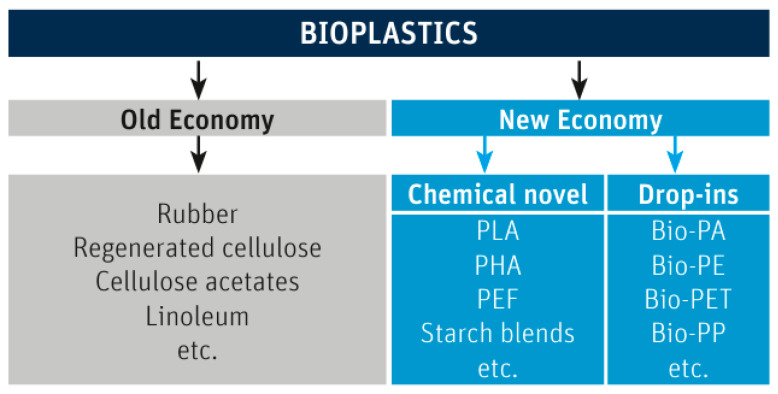
Bioplastics can be grouped into “old” and “new” economy. Reproduced with permission from [9].

A biopolymer is a macromolecule that is composed of biobased or “natural” building blocks. Plastics can be thermoplastics (the largest group), elastomers, or thermosets, and bioplastics can fall into any of these groups. Sometimes, the terms “biopolymer” and “bioplastics” are used synonymously; however, we prefer a delineation with the term “bioplastics” being used for the human-made product (formulation, compound) of biopolymer + other ingredients, for use in technical applications (processing and manufacturing of goods). “Bioplastics” are either biobased and/or biodegradable, at least to a certain degree and as per a given definition (standard, test method). Figure 2 summarizes the definition of bioplastics by the IfBB (Institute for Bioplastics and Biocomposites, Hannover, Germany) [9].

The “old economy” bioplastics include rubber (used in tires), cellulose acetate (deployed in cigarette filters), and linoleum (found in floor systems). Vehicle tire abrasion, by the way, is one of the major sources of non-degradable microplastics, comparable in amount to fibers from polyester-based clothing [10] and plastics nurdles (pellets). While the sap of the rubber tree (*Hevea brasiliensis*) is biodegradable, the vulcanized natural rubber is cross-linked and persistent in the environment. The “new economy” bioplastics include “drop in” materials, which are essentially classic plastics made from a renewable resource, e.g., PE made from ethanol derived from sugar cane (“bio-PE”). They are biobased and have the advantage of behaving just in the same way as their fossil counterparts, so that converters do not need to change any settings in their manufacturing processes. Such materials are equally recyclable as fossil plastics; however, they undergo the same problematic end-of-life scenario as their fossil-based blueprints do: They are recalcitrant towards biodegradation, and a full life cycle assessment (LCA) is needed to describe and compare environmental impacts [11]. Most notably, they also generate persistent microplastics. The “chemical novel” bioplastics in Figure 2 are bioplastics that have no 1:1 correspondence among fossil plastics, with varying degrees of biobased carbon content and biodegradability. Examples are PLA or polyhydroxyalkanoates (PHAs) [12], which also require their own settings on processing equipment, as will be detailed below.

**Figure 3:**
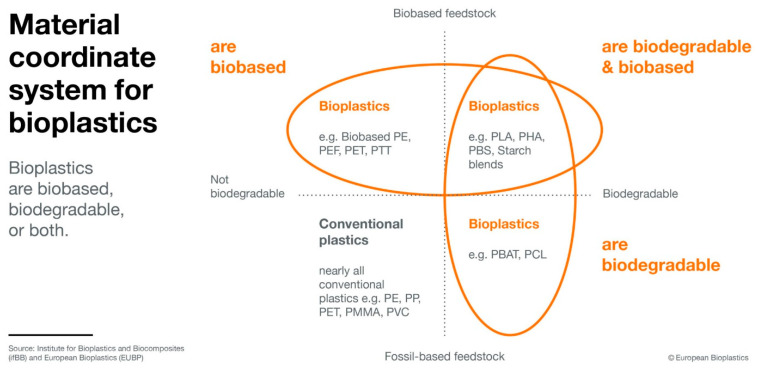
Categories of biodegradable and non-biodegradable plastics. Reproduced with permission from European Bioplastics [13].

Figure 3 depicts bioplastics according to their two main characteristics “biodegradability” and “carbon source”.

Bioplastics are found in the blue and green boxes of Figure 3, not only in their overlapping region. They do not need to be biodegradable, and neither do they need to be biobased, as long as the other criterion is fulfilled. They can be produced by microorganisms or by chemical synthesis, from inorganic (e.g., CO_2_) or organic (e.g., CH_4_, sugar, starch) raw materials. Plants might be used as hosts, too. Apart from plant-based raw materials, animal-derived waste products (e.g., chitin) can be deployed.

Common bioplastics are summarized in the following Table 1 (note that in practice, blends are often used).

PLA is one of the most commonly used and best-established bioplastic materials.

PHAs (polyhydroxyalkanoates) are a class of biopolymers, where the most common representatives are the homopolyesters poly(3-hydroxybutyrate) (P3HB), poly(4-hydroxybutyrate) (P4HB), and, to a lesser extent, poly(3-hydroxyvalerate) (PHV), along with their copolymers poly(3-hydroxybutyrate-*co*-3-hydroxyvalerate) (PHBV) and poly(3-hydroxybutyrate-*co*-3-hydroxyhexanoate) (PHBHx), medium-chain-length PHAs like PHO (poly(3-hydroxyoctanoate)) homopolyesters, and their copolymers and blends [8]. An emerging field in PHA development concerns mcl-PHAs (medium-chain-length PHAs), which display properties of elastomers and bio-latexes [23].

Other bioplastics of lower volumes are, e.g., poly(glycolic acid) (PGA) and the copolymer of glycolic acid and lactic acid (PLGA), PPC (poly(propylene carbonate)) and PFA (poly(furfuryl alcohol)), chitosan, and protein-based (e.g., whey retentate-based) bioplastics. Most bioplastics are thermoplastics. An example of a degradable thermoset is the product made from citric acid + glycerol [24].

Bioplastics, analogous to conventional plastics, can also contain organic fillers, like wood chips or wood dust (WPC, wood—plastic composite), paper fibers, and natural fibers like kenaf, sisal, or hemp [25], which enable them to be fully biobased and biodegradable. Natural inorganic fillers, such as nanoclays [26], which trigger specific material properties like gas barrier behavior, are feasible, too.

Below, in Table 2, several definitions from SAPEA (Science Advice for Policy by European Academies) [27] related to the field of biopolymers and bioplastics are provided.

The terms “biodegradable” and “biobased” will be revisited later in this manuscript in more depth. For a good primer on bioplastics, see, e.g., references [28,29].

### 2.2. Greenwashing

With environmental problems becoming visible and a growing concern for the general public, organizations are starting to feel more pressure to justify their actions and to prove their good-doing, in an attempt to secure or enlarge their business. Consumers have become eco-anxious and spend money on more costly, supposedly more environmentally benign merchandise. Corporate social responsibility (CSR) has become a buzz term in this respect, where organizations address social and environmental concerns in their business operations on a voluntary basis. An organization or its individuals might become tempted to use marketing spins to present themselves as “eco”, “green”, or “good” to the outside world. The expression of “greenwashing” describes dishonest practices of organizations to appear “green” [30]. It purportedly was coined in 1986 by Jay Westervelt, an environmentalist, who observed hotels’ notices encouraging guests to reuse towels, while at the same time harming the environment in stronger ways, which he felt was obscured by directing peoples’ attention to a lesser point of concern. Greenwashing is being blamed, e.g., by environmental groups, but still very present. For a systematic review on concepts and forms of greenwashing, see, e.g., [31]. The trading of carbon emissions can also fall into the realm of greenwashing, when “free credits” are allocated to large emitters, the carbon price is low, carbon projects are only temporary or miscalculated, or consumers feel “clean” after having bought voluntary credits, while continuing with carbon-intense patterns. In addition, it needs to be stated that only a fraction of anthropogenic CO_2_, CH_4_, and other GHG (greenhouse gases) is covered by emissions trading and related schemes. The credibility of the various certificates for renewable energy and circular/sustainable materials can differ among the plentiful, sometimes non-accredited schemes. For a definition of circularity/circular economy, see [32]. One has to acknowledge that the industry is still developing, yet rigid and traceable standards are imperative right from the start, and realistic assumptions particularly for the mid and long term are vital. The expression “carbon footprint”, by the way, was invented by British Petroleum back in 2005 as a marketing sham [33]; they reframed the fossil fuel industry’s responsibility for CO_2_ emissions as consumers’ very own responsibility or “problem”, by asking them about “their carbon footprint”. The industry is not in the spotlight when this term is being used and applied, e.g., via various “CO_2_ footprint calculation” tools. Hence, we should avoid blindly repeating marketing speak with the term “carbon footprint”, and reframe that to the “fossil fuel footprint”. The responsibility of consumers with regards to environmental harm exists, yet we must not overemphasize it or put all of the blame/burden on their shoulders. It is the legal framework in which market incumbents operate and decide to place products on the market that is more the culprit. The consumers, in the end, can only chose among what they are being offered. Lately, a lot of products have been placed on the market with claims related to sustainability, which give the impression of being “eco-friendly”, yet we need true solutions to the plastic waste crisis that have to come from the materials side. It is obvious that “end of pipe” solutions of more waste collection, sorting, and recycling cannot completely solve the problem of persistent plastic waste in nature, as there will always be a certain rate of leakage, both of bulk items as well as of micro- and nanoplastics. Also, the use of additives in plastic formulations needs to be watched carefully, with full transparency and limitations on problematic ingredients.

### 2.3. Biodegradability and Biobased Carbon as Complete Solution

Eventually, fossil plastics, with their stable carbon–carbon backbones, will degrade (in the order of up to hundreds of years), and all fossil carbon was once living matter (millions of years ago). Absolute statements have to be treated with caution, as with the degree to which different materials can be compared to one another, like in the case of, e.g., the toxicity of certain compounds. There is no such thing as a clear definition of “biodegradability” because that property is multifaceted. Let us draw an analogy to woody biomass: A large stem of a tree will take years, or even decades, to “disappear”, while leaves will be biodegraded within less than one year; the same is valid for the stem when undergoing crushing processes prior to biodegradation. [27] states: “*We consider plastic biodegradation a system property, in that it results from the interplay of a specific material property of the plastic that makes it potentially biodegradable as well as the abiotic and biotic conditions in the specific receiving environment that leverage this potential and control the rates and extents of actual plastic biodegradation*”.

The biodegradation of plastics is believed to progress in two steps, which can be preceded and accompanied by mechanical fragmentation (see also [34]):

(1) Breakdown of the polymeric macromolecules into low-molecular-weight moieties.
(2) Uptake of these compounds by microorganisms and in metabolic consumption, to finally yield CO_2_, CH_4_, and H_2_O (complete mineralization).

The mere (bio)degradability of plastics is seen as insufficient to solve the plastic waste problem that the world is facing today because that property might tempt people to neglect the waste hierarchy and to use the materials in a linear fashion alone, with large quantities of plastic waste being littered/mismanaged. In 2020, the EU Group of Chief Scientific Advisors wrote regarding biodegradable plastics: “*The 2018 EU Plastics Strategy sets out a cautious approach for the use of biodegradable plastics (BDP). While it acknowledges that targeted BDP applications have shown some benefits, it also identifies several challenges and points out that “It is important to ensure that consumers are provided with clear and correct information, and to make sure that biodegradable plastics are not put forward as a solution to littering*” [35]. They hence recommend that we should “*limit the use of BDPs in the open environment to specific applications for which reduction, reuse, and recycling are not feasible*”. The nova-Institute has identified such applications in its study BioSinn [36], which are listed in an exemplary fashion here in Table 3.

Examples are plastic components in fireworks, fruit and vegetable stickers, floral foam, dolly ropes, and wet wipes, where reuse and recycling of the materials is hardly feasible. While many packaging and other applications allow up- and downcycling of the materials, there are use cases like those presented in Table 3 above where a substantial fraction of a product will end up in the open environment and cannot be collected. Still, degradability makes sense for many more products, when one considers the considerable fraction of leaked plastics (bulk items and microplastics [37]), e.g., wrappings of sweets and small snacks, golf tees, and various single-use items (where no suitable alternative exists, e.g., a wound dressing). Primary microplastics stem from textiles (which are often made from PTT, PET, or poly(tetrafluoroethylene) (PTFE)—Gore-Tex™) and tire abrasion, which is considered one of the key sources of secondary microplastic [38], and even abrasion of shoe soles [39]. Paints and coatings are another important source of plastic-containing microparticles. Such microplastics are also generated without any littering, both in the use phase and during recycling, so degradability of plastics will, in any case, be beneficial to avoid accumulation and build-up of such materials in the environment. Degradable plastics can mitigate the consequences of both primary and secondary microplastics. Microplastics from degradable polymer materials can, however, also have detrimental effects on the environment, e.g., when that additional carbon freight is brought to an ecosystem or the particles act as carriers for absorbed toxins [40]. A combined approach is needed, where the freight of plastics ending up in the open environment is significantly reduced, and where that material will mineralize as soon as possible. Depending on the particles’ size and type of biodegradable plastics, as well as the ecosystem, the degradation can last from days to years, but not hundreds of years as in the reference case with the xenobiotic fossil plastics.

## 3. It’s All about the Carbon: Renewable Carbon Views and Concepts

Products require energy and raw materials for their manufacturing. The energy can be renewable or fossil, and the same principle applies to materials. Biomass is an example of a classic renewable resource. However, this needs to be regarded in a differentiated manner. When, for instance, an “old” ecosystem is looted to obtain biomass for combustion or material use, it will take a long time (i.e., in the order of at least decades) until an equivalent biomass has regrown, such as via reforestation; so, there can be a justified doubt about true circularity. A Norwegian study [41] found that “*increasing the use of wood from a boreal forest to replace coal in power stations will create a carbon debt that will only be repaid after almost two centuries of regrowth*”. Negative environmental impacts of direct and indirect land use change, for instance, are also well-established, and the use of primary agricultural products for materials can bear negative consequences for feed and food prices, water, land, and fertilizer usage; hence, biomass by itself is not automatically a solution to all fossil energy carriers. While it is clear that carbon from fossil resources is not renewable, there is a debate on carbon from waste streams. Waste incineration (waste-to-energy), for instance, is sometimes promoted as renewable energy, despite the fact that waste can and does contain substantial amounts of fossil carbon and is a carbon-intense process. Through incineration, waste streams are rendered inert and reduced in volume by the process, and energy is obtained, but there will still be a net CO_2_ addition to the atmosphere. What is needed is sustainably sourced, scalable biomass, preferably waste streams from a local source. The key message to take away from this mindset is the following: Our materials need to be defossilized instead of decarbonized.

### 3.1. The Carbon Circle

Carbon is literally the backbone of organic materials and life, and there is a continuous cycle of carbon [42] on Earth, one that has been disrupted by mankind in the last 300 years due to the introduction of carbon, mainly via CO_2_ and CH_4_, from large sinks in the geosphere to the atmosphere, where an unprecedented rate of rise has been observed, currently standing at above 400 ppm of CO_2_. Human activities lead to an imbalance, and fossil resources (coal, oil, natural gas) that are burnt or converted into materials bring back “old” sequestered carbon into the environment. It is estimated that in Europe, 4–6% of all consumed oil and gas is converted into plastics [43]. With landfilling bans becoming effective, a large fraction of these plastics, most of which are short-lived products, is incinerated and converted to CO_2_.

It has proven advantageous to distinguish between carbon from fossil resources that is “dumped” into the atmosphere and carbon that is being reused so that it can be regarded as “renewable”. According to the nova-Institute, the following definition applies: “*Renewable carbon entails all carbon sources that avoid or substitute the use of any additional fossil carbon from the geosphere. Renewable carbon can come from the biosphere, atmosphere or technosphere—but not from the geosphere. Renewable carbon circulates between biosphere, atmosphere or technosphere, creating a carbon circular economy*” [44].

### 3.2. A Side Step at Renewable Energy—Is There a Modern Equivalent to Sales of Indulgences?

Typically, an energy producer will utilize a mix of technologies to provide electricity to its customers, from caloric power stations running on fossil fuels to renewable electricity from, e.g., photovoltaics, wind energy, or hydropower. The share of fossil fuels in the global energy mix has been around 80% for decades and is projected to reduce to 75% by 2030 according to the IEA World Energy Outlook (WEO 2022) [45].

The energy producer can calculate its average CO_2_ footprint in “kg of CO_2_ per kWh of electricity sold” and communicate that number to interested parties. It can also decide to sell the fraction of its “green” electricity to eco-minded customers, who can then claim to be using “100% of renewable” and/or “100% emission-free” electricity (compare the initiatives of scope I, II, and III carbon reporting in annual reports and CSR documents of large corporations, where legal requirements start kicking in). Power has no tag attached to it, so the “green” electricity is actually from the mix of power generation sources, yet the allocation (see “Energy Attribute Certificates” (EACs)) makes some higher-paying customers feel at peace as they are assured they are using carbon-free power. An analogy to the medieval sale of indulgences by the Roman Catholic church comes to mind. Speaking in favor of the practice, one can argue that more demand for “green” electricity will drive the market towards more production of the same in the mid and long run.

In a similar concept, “renewable” carbon or “biobased” carbon can be “attributed” or ascribed to products, giving birth to the term “bio-attributed plastics”. There are standards for such practices, e.g., the ISCC PLUS Version 3.4 scheme ([46] and also see below).

### 3.3. Biobased

When a renewable feedstock is used to make a bioplastic material, it will be “biobased”—at least partly. The term “biobased” is used interchangeably with “renewable” here. Renewable carbon does not necessarily have to be biobased. Direct air capture of CO_2_ and subsequent electrochemical conversion could be performed to obtain the monomers for a bioplastic material; however, there is no such commercial process implemented yet. One could derive the degree of “biobasedness” from the mass of the respective raw materials. For instance, in a compound of 30% (weight) starch in PE, one could argue that 30% of the material is biobased (renewable). However, starch contains moisture, and its formula (C_6_H_10_O_5_)_n_ differs from that of PE (C_2_H_4_)_m_. On a mole basis, only 22% of this compound is organic carbon in this example. The organic carbon content is determined by the radiocarbon method according to ASTM D6866 (“Standard Test Methods for Determining the Biobased Content of Solid, Liquid, and Gaseous Samples Using Radiocarbon Analysis”) [47]. The radiocarbon method quantifies the isotope ^14^C, which has a half-life of 5730 years, and allows the age determination of archeologic artefacts up to approx. 60,000 years, the quality control of food [48], and the determination of biobased carbon content, since fossil carbon is devoid of ^14^C. Alternative standards to ATSM D6866, which is used most frequently in the bioplastics industry today, are ISO 16620-2:2019 “Plastics—Biobased content—Part 2: Determination of biobased carbon content” [49] and EN 16640 “Bio-based products—Bio-based carbon content—Determination of the bio-based carbon content using the radiocarbon method” [50,51]. Inorganic carbon would be black carbon or carbonates used as fillers in plastics, for instance.

For its certification as “OK biobased”, TÜV Austria moves from ASTM D6866 to EN 16640 [52]. The certified products, which can be resins, intermediate products, and final articles, are classified with one to four stars to indicate the range of biobased carbon content. This gives consumers guidance and is partly linked to legal requirements, e.g., in the case of single-use plastics bags under many jurisdictions.

### 3.4. Bio-Attributed

The term “bio-attributed” is rather new and marketed, e.g., for PVC [53] and styrenics [54] like acrylonitrile butadiene styrene [55] (ABS), as well as polyoxymethylene and polyacetal [56] (POM). In the transition towards an increased biobased carbon content, raw materials of biological and fossil origins are mixed in the production process, and by mass-balancing the biobased carbon content is calculated and called “bio-attributed”. Synonymously used terms are “bio-balanced” or “biomass-balanced”. Several products bear claims like up to “x% plant-based”, for instance, PET bottles with biobased ethene glycol (“Plantbottle”™), where that percentage will hardly be reached in real-life products since the material is mixed with purely or mostly fossil-based recycled PET (see ISO 14021:2016 “Environmental labels and declarations—Self-declared environmental claims” [57]). The Eco Mark Office of the Japan Environment Association has recently issued guidelines on “bio-attributed plastics” in its certification scheme [58].

## 4. Degradability of Plastics in General

Plastics are attacked by UV light, which can make the material brittle and easy to disintegrate under mechanical stress. UV can cleave bonds and thereby degrade the plastics, until at an endpoint, full mineralization occurs. High temperature also leads to degradation of plastics, where, e.g., mechanical recycling can create degradation products that might be toxic, and the number of “recycling rounds” can be limited as chain scissions lead to a loss of mechanical properties. Time scales of degradation of plastic products in nature can be estimated at up to hundreds of years for conventional plastics like PE, PP, and PVC. Below, several concepts of degradability are described. Thermal or thermocatalytic degradation processes aim at depolymerization (a.k.a. feedstock recycling), where ideally the monomers are won back. The concept holds promise for thermoplastics, elastomers, and thermosets. Several research projects have studied pyrolysis of plastic waste for recycling purposes. Biodegradable plastics are cleaved enzymatically in various environments, at times scales in the order of months to years, comparable to other natural organic materials. Water-soluble polymers like poly(vinyl alcohol) (PVA) cannot be considered biodegradable since the monomers will exhibit comparatively long residence times in nature. Such films, in order to be truly biodegradable, would need to be made from naturally occurring materials such as alginate.

A misconception or even prejudice towards bioplastics seems to sometimes persist with consumers, in that there is a belief that bioplastics will decompose by themselves within a few months to years while still in use, rendering them intrinsically inferior to fossil plastics in performance. This is of course not the case; the shelf life of bioplastic pellets depends on storage conditions and is, in principle, the same as for fossil plastics, as evidenced by an analysis of technical data sheets of bioplastic producers vs. fossil plastic manufacturers. When, e.g., wood, paper, or any other natural material is kept in a dry place (indoors), it does not auto-decompose either. That analogy can be applied to bioplastic products with biodegradable properties, such as PHA-based shampoo bottles stored in the bathroom—they will not disintegrate there! It is only when they come in contact with microorganisms in the environment that they start degrading. Plastic products “falling apart” only happens under UV light irradiation, in general, and with oxo-degradable materials—see the following paragraph.

### 4.1. Oxo-Degradable

So-called “oxo-degradable” or oxo-fragmentable plastics are an outdated concept of truly single-use plastics, where additives bring about a fragmentation of the material—typically conventional fossil and non-degradable plastics—into microplastic particles within a defined, short period of time. The material is not truly degraded (and absolutely not bio-degraded), but simply disintegrated. Such materials should neither be promoted nor used any further [59]. In the EU, the single-use plastics directive (SUPD, Directive EU 2019/904) [60] has banned oxo-degradable products since 2021. They are an example of misleading consumers and harming the environment, through the introduction of reactive microplastic particles. “Out of sight” for the naked eye clearly does not mean degraded, and we are faced with persistent microplastics in almost all ecosystems, e.g., agricultural soils and water bodies, stemming from conventional, fragmented plastics, but also such oxo-degradable materials.

### 4.2. (Bio)Degradable

Most (biodegradable) bioplastics are polyesters, and as such, they can be hydrolyzed by hydrolytic esterase enzymes. Under the action of microorganisms (bacteria [61] and fungi [62]), plastics can be degraded by enzymatic cleavage of the polymer chains. Natural polymers are generally biodegradable (unless they are crosslinked, like is the case in, e.g., vulcanized natural rubber). Also, several synthetic polymers can be biodegraded, e.g., the polyester poly(ε-caprolactone) (PCL). Since enzymatic activity by microorganisms is stronger in (hot and humid) compost than in soil, and even less in freshwater and the least in cold sea water, the environment plays a critical role in the rate of (bio)degradability. Depending on the availability of oxygen, aerobic and anaerobic degradation mechanisms occur. In general, when water can access the material, degradability tends to be faster [63]. Also, amorphous plastics degrade faster than crystalline ones, as was demonstrated for, e.g., bio-based poly(butylene adipate-*co*-butylene furandicarboxylate) [64] or for poly(lactic-*co*-glycolic acid) (PLGA) [65]. The degradability of compounds and blends is influenced by the degradability of the individual components. Thin (and foamed) products naturally degrade faster than bulky items. The presence of additives can influence biodegradability rates, too. Biodegradation under aerobic conditions differs from anaerobic processes, where CH_4_ plus CO_2_ are typically formed. In this case, the impact on the climate needs to be considered because of the emission of that CH_4_. In recent years, several test methods and standards, plus their certifications and labels, were developed to define biodegradability under certain environmental conditions, like, e.g., “marine degradability”, “home compostability”, or “industrial compostability”. These will be summarized below.

The nova-Institute distinguishes six relevant settings for biodegradability [36]:

(1) Industrial composting;
(2) Home composting;
(3) Soil;
(4) Wastewater;
(5) Freshwater;
(6) Salt (marine) water.

European Bioplastics (EU BP) has summarized the most relevant standards for biodegradability [66]. For a summary of standards and labels, see also [67]. Normec OWS, a large test provider of biodegradability, offers tests in various environments, e.g., according to standards ASTM D6400, ASTM D6868, ASTM D8410, AS 4736, EN 14995, EN 13432, ISO 17088, and ISO 18606, with the possibility for certificates under several jurisdictions:

- Australia: ABA (Seedling);
- Europe: DIN CERTO (Seedling, DIN Geprüft Industrial Compostable, DINPlus) and TÜV AUSTRIA (OK Compost, Seedling);
- Japan: JBPA (GreenPla) [68];
- USA: BPI (Compostable).

TÜV Austria provides these certifications, as introduced by Vinçotte:

- OK biodegradable MARINE (requirements equivalent to ASTM D 6691, 6 months);
- OK biodegradable SOIL (requirements equivalent to DIN EN 13432 but not 6 months, instead 24 months);
- OK biodegradable WATER (requirements equivalent to DIN EN 13432, but max. 25 °C and 56 days);
- OK compost INDUSTRIAL (requirements equivalent to DIN EN 13432);
- OK compost HOME (requirements equivalent to DIN EN 13432, max. 30 °C).

Apart from actual degradability, the products to be certified must be intended for use in these specific environments (not by littering). Another well-known certification body is DIN CERTCO (TÜV Rheinland).

For “industrial compostability” certification, Normec OWS demands disintegration and biodegradability as follows:

- A 90% disintegration within 12 weeks;
- A 90% carbon-to-CO_2_ conversion at 58 °C within 6 months.

These biodegradation tests are based on ISO 14855, ASTM D 5338, and/or EN 14046, and on ISO 16929 and/or EN 14045 for disintegration [68]. Ecotoxicity can be assessed according to the OECD 208 test method.

Biodegradability is a complex topic, with several partly overlapping and partly diverging standards, superimposed by blurred terminology and concepts. Not all certificates are issued by accredited organizations, and sometimes misinformation or lack of knowledge leads to inaccurate statements. With evolving legal regulations stipulating minimum requirements for biodegradability, as well as biobased carbon content, precise definitions and unambiguous tests are imperative. Below, in Table 4, several important standards are summarized with regards to biodegradability.

A bioplastic may be biodegradable, but not compostable, when its kinetics are, e.g., too slow to meet the metrics of the compostability standard (home and industrial). Some materials need the high temperature of composting (58 °C) for timely degradation, so they can be classified “industrial compostable” but not degradable in the open environment, particularly in difficult environments like marine systems, e.g., PLA.

A common logo for compostability is the “seedling”. It is used to show “compostability” based on EN 13432, and it is acknowledged in Belgium, Switzerland, Germany, The Netherlands, Poland, the United Kingdom, and other countries [104]. It needs to be stated that even though several bioplastics meet the criteria of being compostable, they may still be partly visible at the end of a composting process, because industrial composting plants often have very short residence times of several weeks only, though even thin materials will not be mineralized that fast.

The test schemes summarized above are applied to the certification of specific polymers, as well as plastics (i.e., compounds with blend partners and/or additives) and finished items (e.g., a film or an injection-molded consumer good).

Note that commonly only 90% degradation is demanded (e.g., by EN 13432), allowing for the use of conventional, non-degradable additives.

### 4.3. Controlled Degradability (Enzyme Mediated)

For some products, a set lifetime is desirable. For instance, mulching films should last one season, whereas a plastic clip to protect young trees from deer would typically be needed during the first 3 years of the plants’ life. The “lifetime” of a given bioplastic product can be influenced by its composition (blend of different biopolymers) and by the addition (or absence) of certain additives, e.g., enzymes for controlled degradability. Enzyme-mediated bioplastics are an emerging field of research, see, e.g., [105,106].

### 4.4. Recyclable Plastics and the Definition of “Renewable” and “Circular”

Renewable raw materials (biomass) can be converted into renewable plastics. When plastics are recycled, they can be called “circular”. Sometimes, stakeholders also use the term “renewable” or “renewed” interchangeably and synonymously with “recycled” and “recyclable” when referring to such materials. Plastics recycling is a key pillar of a circular economy, and as such, desirable. Care has to be taken about possible microplastic emissions and energy consumption, i.e., a full life cycle assessment is required to ensure that the recycling process makes sense. In general, bioplastics are recyclable. “Thermal recycling”, a.k.a. waste incineration, is a misnomer when it is applied to plastics after single use, but can be useful in a cascaded concept when the energy is captured—but CO_2_ is being released to the atmosphere. For bioplastics to be truly “sustainable plastics”, their end-of-life-scenarios need to be considered (see Table 5 [27]).

As Table 5 illustrates, bioplastics are not automatically compatible with collection and recycling schemes of conventional plastics. For instance, there is the fear that a certain fraction of bioplastics in PET could lead to deterioration of the properties in recycling, potentially making injection blow molding of recycled material unfeasible. This is a “hen and egg” problem, and solutions need to be developed for collecting, sorting, and recycling more types of plastics “post-consumer”, which hardly exist today for any other plastic products than PET bottles. While many plastics are recycled in closed loops in industrial settings, e.g., the gatings (sprues) in injection molding, post-consumer plastic waste is very difficult to recycle due to the variety of products, difficulties in separation, and low volumes per grade. In as much as composite materials allow a perfect tailoring of material properties, they make recycling difficult, and we are far from full life cycle design and material standardization.

## 5. Discussion

Factors that limit the widespread use of bioplastics are their lack of availability, missing experience, absence of incentives/legal framework, and costs. The costs of bioplastics are higher, in general, than those of fossil plastics, leaving externalized costs aside. The cost drivers are smaller economies of scale (the biobased industry tends to operate at smaller scale than the petrochemical one), less industrial maturity (while petrochemical refineries are fully integrated, biorefineries often have a limited number of final products, with side streams that are not fully valorized), higher downstream processing costs, e.g., for intracellular PHA biopolyesters, and more costly feedstock, leading to higher costs/kg of resin. Furthermore, the density of bioplastics tends to be 20–30% higher than for polyolefins, which adds to the costs of the final parts. Some of the cost disadvantages can be overcome by mixing bioplastics with low-density biogenic side streams/waste materials, such as wood dust or fibers [25,107], which results in readily biodegradable composite materials [108]. Moreover, bioplastics are more temperature sensitive than their fossil counterparts, which translates into a smaller processing window with typically lower productivities and thereby again higher unit production costs. However, in the mid to long run, bioplastics need to replace fossil plastics in more and more applications, which they can do already today. We see the challenges of recycling fossil plastics daily, now standing at 9% globally after decades of struggling. Even if all fossil plastics parts were recycled, there would still be the microplastic problems and the fossil carbon footprint.

A reduction of plastic consumption is the first step to be considered, and there are high-risk and unnecessary, very short-lived, single-use products, which should not just be made from biodegradable plastics but be phased out for good, e.g. some excessive packaging, e-cigarettes or cosmetic microbeads. Yet, the demand for plastic materials is there and is steadily growing; the world population is increasing and developing, pushing the desire for products. Moreover, polymer-based formulations are needed in many applications, where other biobased substitutes are neither available nor practical.

**Figure 4:**
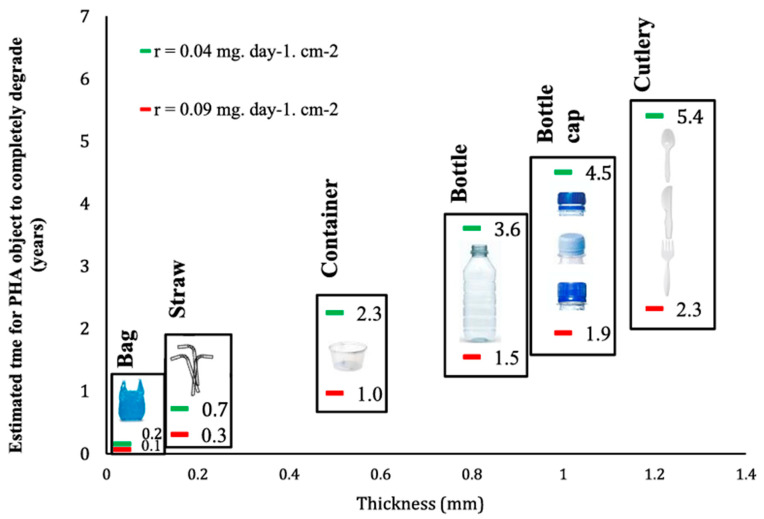
Degradation times of PHA articles in the marine environment. Reproduced with permission from [109].

Bioplastics can help decarbonize/defossilize and circularize the plastic economy, and scientifically proven and honest claims (statements) about their properties need to be made, particularly with regard to being “biobased”, “(bio)degradable”, and possibly also “bio-attributed”, to avoid greenwashing. The concepts, as laid out in this article, are not “black and white”, but need to be regarded in a differentiated manner. The actual biodegradability of a bioplastic-made item depends not only on the polymer but also on the product (e.g., thickness, shape, crystallinity, hydrophobicity), as well as the environment with its ambient conditions, as exemplified in Figure 4 below [109].

The degradability is also influenced by blend partners and additives. As bioplastics are being used more widely—and this trend is expected to continue—clear definitions are important. The controlled degradability of bioplastics, e.g., by adding enzymes, is a highly interesting and promising field for future research, to develop formulations with tailored life times. The demand for such materials arises, e.g., from single-use articles in agriculture, but might possibly also contribute to rendering litter less harmful and less persistent.

The concept of **renewability** is clearly linked with the carbon source, where biomass is the most common raw material. Side streams and waste streams of biomass are advantageous, and other carbon sources such as non-biomass organic waste, and/or CO_2_ from point sources or captured from the atmosphere, are also feasible. Companies that work on converting gaseous feedstocks into PHA are LanzaTech (CO, CO_2_) [109], Mango Materials, Circe Biotechnologie, and Newlight Technologies (CH_4_). In a word, renewability is about circular carbon.

**Biosynthesis** has a clear definition, standing for the fact that a material has been made by plants, animals, bacteria, fungi, algae, or archaea, either naturally occurring ones or genetically modified organisms (GMOs), or by their biocatalytically active parts (enzymes).

**Biodegradability** refers to the enzymatic destruction (catabolism) of polymeric substances by living organisms, intra- or extracellularly, in the environment. A bioprocess with extracted enzymes, for instance, would not fall under the strict definition.

The term “**degradability**” includes all means, even radiation-mediated and thermal processes, which might occur in nature (abiotically) or in technical systems. Typically, when referring to the degradability of plastics or bioplastics, biodegradability is to be understood.

Finally, a **biocompatible** material is one that does not exert toxic or injurious effects on biological systems, a property borne by several fossil plastic materials and not necessarily being an inherent feature of biobased polymers. This concept has applications in the medical area, for instance [110].

The use of renewable resources can, therefore, be considered an important aspect [111], but it does not entirely solve the plastics crisis we are in today. We need a differentiated view on degradability/biodegradability. In this review, approx. 50 existing standards for determining biodegradability and biobased carbon content were compiled. They are partly overlapping and require specialized test institutes to perform assessments against them. Previously, attempts were made to estimate biodegradability rates based on gas evolution or differential scanning calorimetry (DSC) changes. Since biological systems are complex, tests need to be standardized and carefully be described, to ensure reproducibility. At present, there is no universal “quick test” for biodegradability, and it is likely that there will not be any such solution in the future. The selection of a test method that is relevant and most appropriate should be based on the target application and possible end-of-life scenarios of any given bioplastic product. In any case, responsible use, particularly at the end of the life cycle, and recycling should be fostered, including bioplastic materials, with biodegradation being a possible, but not the preferred solution for the products in question.

## 6. Conclusions

In the field of bioplastics, there are clear and unambiguous definitions of, e.g., the properties of being biobased, bio-attributed, and biodegradable, and stakeholders should use these terms in their exact meanings.

A “golden rule” in material selection is that there is seldom a clear “right” or “wrong”; instead, several materials can typically fulfil the requirements of a given applications. Fossil plastics can do the job in many cases, but they need to be managed well. Even then, they can still have detrimental effects on the environment, e.g., through microplastics’ and nanoplastics’ formation during the use or recycling phases, and at the end of their lifetime, typically they add to anthropogenic CO_2_ emissions. The definition of “bioplastics” should be narrowed to materials that are both biobased and—with details depending on the type of material and the application/environment—intrinsically biodegradable, as well as free from problematic additives. We should prefer additives approved for food contact materials as well as those that are biobased and biodegradable. Since micro- and nanoplastics from virtually all products can enter the food chain, more care should be taken in their formulation and development in terms of product life cycle management. Bioplastic materials meeting the definition *sensu stricto* are an intrinsically better solution than conventional plastics because they are less harmful when being “lost” inadvertently. In these cases, they degrade as microplastics and bulk materials in a “reasonable” time, in the order of at most years instead of centuries. Apart from the standards for biobased carbon content and biodegradability for different environments, a standard concerning transparency and selection of additives is needed, to provide full clarity throughout the value chain about the formulations. We should intensify efforts to defossilize the plastic industry, replacing conventional material with biobased and biodegradable substitutes, coupled with actions for the reduction of consumption and waste management, and we should ensure that proper definitions are applied to materials to avoid ambiguity. The feedstock for bioplastics should not be primary agricultural products, but side streams and waste streams, since high volumes are needed at full roll-out; this is relevant to the development of the bio-industry from first-generation materials to second-generation solutions.

More research will also be needed to develop advanced additives for bioplastics, not only to replace today’s chemicals but also to formulate ones that are tailored to specific materials, to increase their performance. The emphasis on composites may be on fully biobased compounds, and innovative ideas for recycling including material standardization, labelling, collection, and circularity are needed. Disruptive ideas on reuse of materials will further support efforts to make material usage more sustainable. Clear definitions remain at the center of the transition from fossil to biobased and biodegradable plastics, so that we can keep the strong benefits that plastics have brought to us over the last seven decades and minimize their unwanted side effects.

## Figures and Tables

**Table 1:** Overview of bioplastics. “x” stands for “yes” and “(x)” for “partly”. The number and alignment of arrows in the column “trend” give a qualitative indication on expected market volume development in the coming years (↑ to ↑↑↑↑: noticeable to rapid development; ↗: modest development; →: stagnation).

Bioplastic Material	Short	Family (Class)	Biobased	Biodegradable	Applications	Fossil Counterparts	Market Volume [9], 2022, in kt/a	Trend	Comment
Poly(lactic acid)	PLA	Polyester	x	(x)	Packaging, 3D printing, consumer goods, medical fields, agriculture	PS	430	↑↑	[14]
Polyhydroxyalkanoates	PHA	Polyester	x	x	Packaging, 3D printing, biomedical use, bioremediation, commodity materials	PP and others	93	↑↑↑↑	[15]
Poly(butylene succinate)	PBS	Polyester	(x)	(x)	Packaging, disposable tableware, medical articles, agriculture (mulching films, release of pesticides, and fertilizers), fishery		90	→	[16]
Poly(butylene adipate-*co*-terephthalate)	PBAT	Polyester	(x)	(x)	Packaging, antimicrobial foils, single-use catering items, horti- and agriculture, textile industry	LDPE	310	↗	[17]
Starch, thermoplastic starch	TPS	Polysaccharide	x	x	Injection-molded commodity materials, thermoformable flat films	-	220	→	[18]
Bio-poly(trimethene terephthalate)	Bio-PTT	Drop-in	(x)		Textile fibers (carpets, car floor mats)	PTT	120	→	[19]
Bio-poly(propene)	Bio-PP		(x)		Automotive parts, electrical devices, concrete additive, textile fibers, plastic bank notes in tropical regions, packaging materials	PP	120	↑↑↑	[19]
Bio-polyamide	Bio-PA		(x)		Textile fibers, sailing, parachute, ropes, fishery, horticulture (grass trimmer lines), tennis rackets strings, musical instrument strings	PA	205	↗	[19]
Bio-poly(ethene)	Bio-PE		(x)		Packaging, agriculture, foils, injection-molded parts	PE	300	↑↑	[19]
Bio-poly(ethene terephthalate)	Bio-PET		(x)		Packaging, bottles, foils, textile fibers	PET	100	↗	[19]
Poly(ε-caprolactone)	PCL	Polyester		x	Biomedical use (release of pharmaceuticals, wound glues, tissue engineering), packaging	-			[20]
Cellulose acetate	CA	Polysaccharide (esterified)	x	(x)	Cigarette filters, artificial silk, eye glasses frames	-			[21]
Poly(ethene furanoate)	PEF	Polyester	x	(x)	Bottles, foils, fibers	PET	-		[22]

**Table 2:** Concise definitions related to bioplastics.

Biobased plastic(s)	Plastic containing organic carbon of renewable origin from, plant, animal, or microbial sources	[27]
Biodegradable plastic(s)	Biodegradable plastic. A plastic that undergoes biodegradation involving the metabolic utilization of the plastic carbon by microorganisms such as bacteria, fungi, and algae, resulting in the conversion of plastic carbon to CO_2_ (and CH_4_) and microbial biomass	[27]
Biopolymer	A polymer produced by a living organism or isolated parts thereof (enzymes)	[27]
Degradable plastic	A plastic or matrix that can degrade under certain environmental conditions in specific time period, resulting in loss of properties as measured by standard test methods. Degradation of plastic can result either from hydrolysis (hydrolytic degradation), oxidation (oxidative degradation), light (photo degradation), or a combination of these effects (ASTM D883-20a)	[27]
Degradation	Chemical changes in a polymeric material that usually result in undesirable changes in the in-use properties of the material	[27]
Plastic biodegradation	The microbial conversion of all organic constituents in plastic to carbon dioxide, new microbial biomass, and mineral salts under oxic conditions, or to CO_2_, CH_4_, new microbial biomass, and mineral salts under anoxic conditions	[27]
Renewability	The ability of a resource or energy source to be naturally replenished or restored within a reasonable period, making it sustainable for long-term use without being depleted or exhausted. Renewable resources, such as solar energy, wind energy, hydropower, biomass, and geothermal energy, are considered environmentally friendly alternatives to non-renewable resources like fossil fuels, which have limited availability and contribute to environmental issues like climate change	
Biosynthesis	Polymers can be obtained via synthetic methods, e.g., under pressure or with catalysts, or be synthesized in nature, by, e.g., plants (starch) or bacteria (PHA)	
Biodegradability	Degradability can be brought about by irradiation or mechanical forces, whereas biodegradation is the cleavage of (in our case) polymers into smaller moieties, until complete mineralization to CO_2_ and H_2_O. Biodegradability is caused by enzymes from microorganisms	
Biocompatibility	The ability of a material or substance to safely and effectively interact with living tissues or biological systems without causing harm, adverse reactions, or immune responses. In the medical fields, biocompatible materials are essential for various applications, such as implants, medical devices, drug delivery systems, and tissue engineering. Not all bioplastics are biocompatible, and some fossil plastics also show biocompatibility	

**Table 3:** Examples of applications of plastics where biodegradability makes sense. Source: [36].

Applications with an Estimated >30% of Material Being Lost in the Open Environment	Applications with an Estimated >90% of Material Being Lost in the Open Environment	Applications with an Estimated 100% of Material Being Lost in the Open Environment
Plastic components in fireworks Fruit and vegetable stickers Floral foam Microplastics in cosmetics Dolly ropes Wet wipes	Plant fixing clips Leg bands for birds and wild animals Teabags Tree shelters Biowaste bags Non-durable products for fishery and aquaculture	Packaging film for dishwasher tabs Lawn trimmer threads Seed coating Controlled release carrier substances for fertilizer additives Geotextiles

**Table 4:** Common standards for biodegradability of bioplastic materials (selection).

Standard	Wording	Introduced	Status	Test Conditions	Applicable to	Comments	Ref.
DIN EN 13432	Packaging—Requirements for packaging recoverable through composting and biodegradation—Test scheme and evaluation criteria for the final acceptance of packaging	10/2000	Active	Aerobic conditions (composting): 90% degradation within 6 months Anaerobic conditions (biogas plant): 50% degradation within 3 months	Packaging	Very common standard, often applied (wrongly) to other products (e.g., bulky items, where a certificate of a ~100 µm film is used for a tick injection molded article of the same material by another producer)	[69]
DIN EN 14995	Plastics—Evaluation of compostability—Test scheme and specifications	03/2007	Active	Aerobic conditions (composting): 90% degradation within 6 months Anaerobic conditions (biogas plant): 50% degradation within 3 months	Plastics		[70]
DIN EN 17033	Plastics—Biodegradable mulch films for use in agriculture and horticulture—Requirements and test methods	03/2018	Active	A 90% degradation within 24 months, at 20 °C to 28 °C.	Mulching film		[8]
ASTM D6691—17	Standard Test Method for Determining Aerobic Biodegradation of Plastic Materials in the Marine Environment by a Defined Microbial Consortium or Natural Sea Water Inoculum	12/2017	Active	In 10 to 90 days and at a temperature of 30 °C ± 1 °C	Marine environment		[7]
AS 5810—2010	Biodegradable plastics—Biodegradable plastics suitable for home composting	07/2010	Active	A 90% degradation within 12 months. The temperature in the test is 25 ± 5 °C and must not exceed 30 °C	Home composting		[71]
NF T51-800	Plastics—Specifications for plastics suitable for home composting	11/2015	Active	After 180 days, a max. 10% of the initial dry mass will be retained in a sieve at 2 mm mesh size, in addition to 90% degradation within 12 months at a max. of 30 °C	Home composting		[72]
ISO 14855	Determination of the ultimate aerobic biodegradability of plastic materials under controlled composting conditions—Method by analysis of evolved carbon dioxide—Part 1: General method	12/2012	Active				[73]
ASTM D 5338-15	Standard Test Method for Determining Aerobic Biodegradation Of Plastic Materials Under Controlled Composting Conditions, Incorporating Thermophilic Temperatures	2021	Active	Equivalent to ISO 14855			[74]
ASTM D5988-18	Standard Test Method for Determining Aerobic Biodegradation of Plastic Materials in Soil	09/2018	Active				[75]
DIN EN 14046	Packaging—Evaluation of the ultimate aerobic biodegradability of packaging materials under controlled composting conditions—Method by analysis of released carbon dioxide	07/2003	Active				[76]
ISO 16929	Plastics—Determination of the degree of disintegration of plastic materials under defined composting conditions in a pilot-scale test	03/21	Active				[77]
DIN EN 14045	Packaging—Evaluation of the disintegration of packaging materials in practical oriented tests under defined composting conditions	06/2003	Active				[78]
AS 4736-2006	Biodegradable Plastic-Biodegradable Plastics Suitable for Composting and other Microbial Treatment—Australian Capital Territory	2006	Active	You must not provide customers with a plastic shopping bag unless it is made of a biodegradable plastic. A plastic shopping bag is a bag that is made, in whole or in part, of PE with a thickness of less than 35 microns			[79]
ISO 17088	Plastics—Organic recycling—Specifications for compostable plastics	04/2021	Active				[80]
ASTM D6954-18	Standard Guide for Exposing and Testing Plastics that Degrade in the Environment by a Combination of Oxidation and Biodegradation	03/2018	Active				[81]
ASTM D7991-15	Standard Test Method for Determining Aerobic Biodegradation of Plastics Buried in Sandy Marine Sediment under Controlled Laboratory Conditions	03/2022					[82]
ISO 11266	Soil quality—Guidance on laboratory testing for biodegradation of organic chemicals in soil under aerobic conditions.	09/1994	Active		Chemicals		[83]
ISO 20200	Plastics—Determination of the degree of disintegration of plastic materials under simulated composting conditions in a laboratory-scale test						[84]
ASTM D7081-05	Standard Specification for NonFloating Biodegradable Plastics in the Marine Environment		Withdrawn 2014				[85]
ASTM D6400-23	Standard Specification for Labeling of Plastics Designed to be Aerobically Composted in Municipal or Industrial Facilities	03/2023	Active				[86]
ASTM D6868-21	Standard Specification for Labeling of End Items that Incorporate Plastics and Polymers as Coatings or Additives with Paper and Other Substrates Designed to be Aerobically Composted in Municipal or Industrial Facilities	10/2021	Active				[87]
ISO 14851	Determination of the ultimate aerobic biodegradability of plastic materials in an aqueous medium—Method by measuring the oxygen demand in a closed respirometer	03/2019	Active				[88]
ISO 14852:2018	Determination of the ultimate aerobic biodegradability of plastic materials in an aqueous medium—Method by analysis of evolved carbon dioxide	10/2021	Active				[89]
ISO 14853:2016	Determination of the ultimate anaerobic biodegradation of plastic materials in an aqueous system—Method by measurement of biogas production	07/2016	Active				[90]
ISO 14855-1:2012	Determination of the ultimate aerobic biodegradability of plastic materials under controlled composting conditions—Method by analysis of evolved carbon dioxide—Part 1: General method	04/2013	Active				[91]
ISO 14855-2:2018	Determination of the ultimate aerobic biodegradability of plastic materials under controlled composting conditions—Method by analysis of evolved carbon dioxide—Part 2: Gravimetric measurement of carbon dioxide evolved in a laboratory-scale test	12/2018	Active				[92]
ISO 15473	Soil quality—Guidance on laboratory testing for biodegradation of organic chemicals in soil under anaerobic conditions	03/2002	Active		Chemicals		[93]
ISO 17556	Determination of the ultimate aerobic biodegradability of plastic materials in soil by measuring the oxygen demand in a respirometer or the amount of carbon dioxide evolved	09/2019	Active				[94]
ISO 18830	Determination of aerobic biodegradation of non-floating plastic materials in a seawater/sandy sediment interface—Method by measuring the oxygen demand in closed respirometer	08/2016	Active				[95]
ISO 19679	Determination of aerobic biodegradation of non-floating plastic materials in a seawater/sediment interface—Method by analysis of evolved carbon dioxide	11/2020	Active				[96]
ISO 23977	Plastics—Determination of the aerobic biodegradation of plastic materials exposed to seawater—Part 1: Method by analysis of evolved carbon dioxide	11/2020	Active				[97]
ISO/DIS 23832	Plastics—Test method for determination of degradation rate and disintegration degree of plastic materials exposed to marine environmental matrices under laboratory conditions	06/2021	Active				[98]
ISO 23977-2	Plastics—Determination of the aerobic biodegradation of plastic materials exposed to seawater—Part 2: Method by measuring the oxygen demand in closed respirometer	11/2020	Active				[99]
ISO 5430	Plastics—Marine ecotoxicity testing scheme for biodegradable plastic materials—Test methods and requirements	05/2023	Active				[100]
ISO 22526-3	Plastics. Carbon and environmental footprint of biobased plastics—Process carbon footprint, requirements and guidelines for quantification	08/2020					[101]
ISO 22766	Plastics—Determination of the degree of disintegration of plastic materials in marine habitats under real field conditions	03/2020		Current	03/2020		[102]
DIN EN 17427:2022-08	Packaging—Requirements and test scheme for carrier bags suitable for treatment in well-managed home composting installations; German version	08/22	Active				[34]
DIN EN 14987	Plastics—Evaluation of disposability in waste water treatment plants—Test scheme for final acceptance and specification	02/2007	Active				[103]

**Table 5:** Different end-of-life scenarios for bioplastics compared to conventional plastics. Source: [27].

Disposal Scenario	Potential Outcome		
	Positive	Neutral	Negative
Release into a natural environment that has been appropriately considered and evaluated from the design stage	√		
Release into a natural environment that has not been appropriately considered and evaluated from the design stage		√	√
Transfer to an appropriate managed system for biodegradable materials, e.g., industrial composter	√		
Transfer to an inappropriate managed system for biodegradable materials, e.g., recycling streams for conventional polymers such as PE			√
Transfer to a managed system for residual waste		√	

## Data Availability

No data were used for the research described in this article.
